# Evaluating Behavioral Sleep Medicine Knowledge and Skills in Geropsychologists and Trainees: A Needs Assessment

**DOI:** 10.1093/geroni/igaf122.780

**Published:** 2025-12-31

**Authors:** Catherine Ju, Amy Fiske, Michelle Mlinac, Angelica Boeve, Julia Boyle

**Affiliations:** West Virginia University, Morgantown, West Virginia, United States; West Virginia University, Morgantown, West Virginia, United States; VA Boston Healthcare System, Boston, Massachusetts, United States; Minneapolis VA, Minneapolis, Minnesota, United States; VA Boston Healthcare System, Boston, Massachusetts, United States

## Abstract

Older adults are at greater risk for sleep difficulties which may impact their physical health, mental health, and quality of life. Despite this, research suggests that few clinical psychologists have formal sleep-related training. Given the shortage of clinicians specifically trained to work with older adults, the number of providers who feel competent in delivering behavioral treatment for older adults with sleeping problems is likely lacking. The current study aimed to identify gaps in behavioral sleep medicine knowledge and training in geropsychologists and trainees who work with older adults. Geropsychologists and trainees (N = 83) completed an online survey to evaluate gaps in knowledge and skills related to behavioral sleep medicine within geropsychology. The study found that the community had a general understanding of how sleep presents in older adults; however, they were less familiar with the assessment of sleep disorders beyond insomnia. Respondents were most knowledgeable about presentations of insomnia (94%) and sleep disordered breathing (93%) and least familiar with disorders of hypersomnolence and periodic limb movement disorder. Respondents had provided sleep hygiene education and cognitive behavioral therapy for insomnia more than anything else. Interest in learning about more interventions was distributed across multiple treatments, with brief behavioral therapy for insomnia being the highest, followed by exposure relaxation and rescription therapy. Geropsychologists are uniquely positioned to assess and treat sleep disorders in older adults, and our findings suggest the need for more comprehensive training in order to effectively treat sleep disorders in older adults.

